# Cytotoxic data of 14-deoxy-11, 12-didehydroandrographolide (14-DDA), double transfection and DDIT3 silencing data in T-47D breast carcinoma cells

**DOI:** 10.1016/j.dib.2016.04.046

**Published:** 2016-04-23

**Authors:** Heng Kean Tan, Tengku Sifzizul Tengku Muhammad, Mei Lan Tan

**Affiliations:** a Malaysian Institute of Pharmaceuticals & Nutraceuticals, National Institutes of Biotechnology Malaysia (NIBM), Ministry of Science, Technology & Innovation, Halaman Bukit Gambir, Pulau Pinang, Malaysia; b Advanced Medical & Dental Institute, Universiti Sains Malaysia, Pulau Pinang, Malaysia; c Institute of Marine Biotechnology, Universiti Malaysia Terengganu, Kuala Terengganu, Terengganu, Malaysia

**Keywords:** 14-Deoxy-11,12-didehydroandrographolide, Cytotoxic, DDIT3, Transfection

## Abstract

The data presented in this article are related to the research article entitled “14-deoxy-11,12-didehydroandrographolide induces DDIT3-dependent endoplasmic reticulum stress-mediated autophagy in T-47D breast carcinoma cells”, which the mechanistic toxicology properties of 14-deoxy-11,12-didehydroandrographolide (14-DDA) were investigated (Tan et al., 2016 [1]). This article describes the derivation of cytotoxic parameters of 14-DDA, cell viability data after double transfection and DDIT3 silencing in T-47D cells.

**Specifications Table**

**Table:** 

Subject area	*Pharmacology and toxicology*
More specific subject area	*Cell proliferation assay, RT-qPCR and Western Blot analysis*
Type of data	*Figures*
How data was acquired	*Envision 2104 multilabel plate reader (Perkin Elmer, USA), CFX96™ Real-Time PCR Detection System (Bio-Rad Laboratories, USA), ChemiDoc™ XRS Imaging System (Bio-Rad Laboratories, USA) and Quantity One*^®^*1-D Analysis Software (Bio-Rad Laboratories, USA).*
Data format	*Analyzed*
Experimental factors	*T-47D breast carcinoma cells were either treated with 14-deoxy-11,12-didehydroandrographolide or transfected with siRNA and/or plasmid DNA*
Experimental features	*Cytotoxic parameters, the effects of double transfection on cell viability and DDIT3 silencing in T-47D cells were determined*
Data source location	*Universiti Sains Malaysia, Pulau Pinang, Malaysia*
*Malaysian Institute of Pharmaceuticals and Nutraceutical, NIBM, Pulau Pinang, Malaysia*
Data accessibility	*Data is accessible with this article*

**Value of the data**

• The data is beneficial to researchers who are interested in the pharmacology properties of diterpenoids and *Andrographis paniculata* Nees.
• This data set is beneficial to researchers who want to derive cytotoxic parameters of compounds in cell lines.
• The data is helpful to determine the viability of cell lines after double transfection.
• The data is helpful to ensure silencing of both mRNA and protein expressions at suitable time points when cells are transfected with siRNA.

## 1 Data

**Fig. 1:**
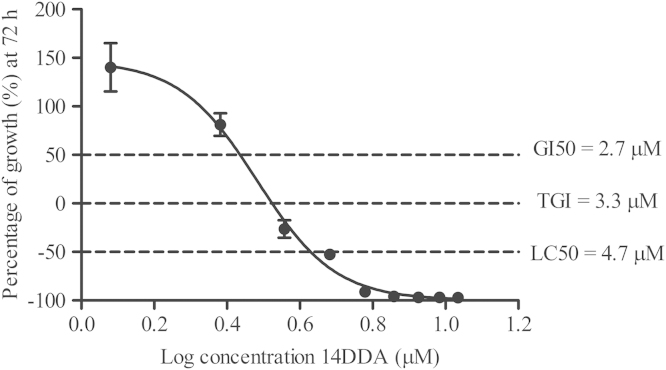
The cytotoxic parameters of 14-DDA in T-47D cells. Positive values (<100%) represent growth inhibition and negative values (<0%) represent cytotoxicity as compared with initial cells plated (T_0_). Data are presented as mean±SD of two independent experiments.

**Fig. 2:**
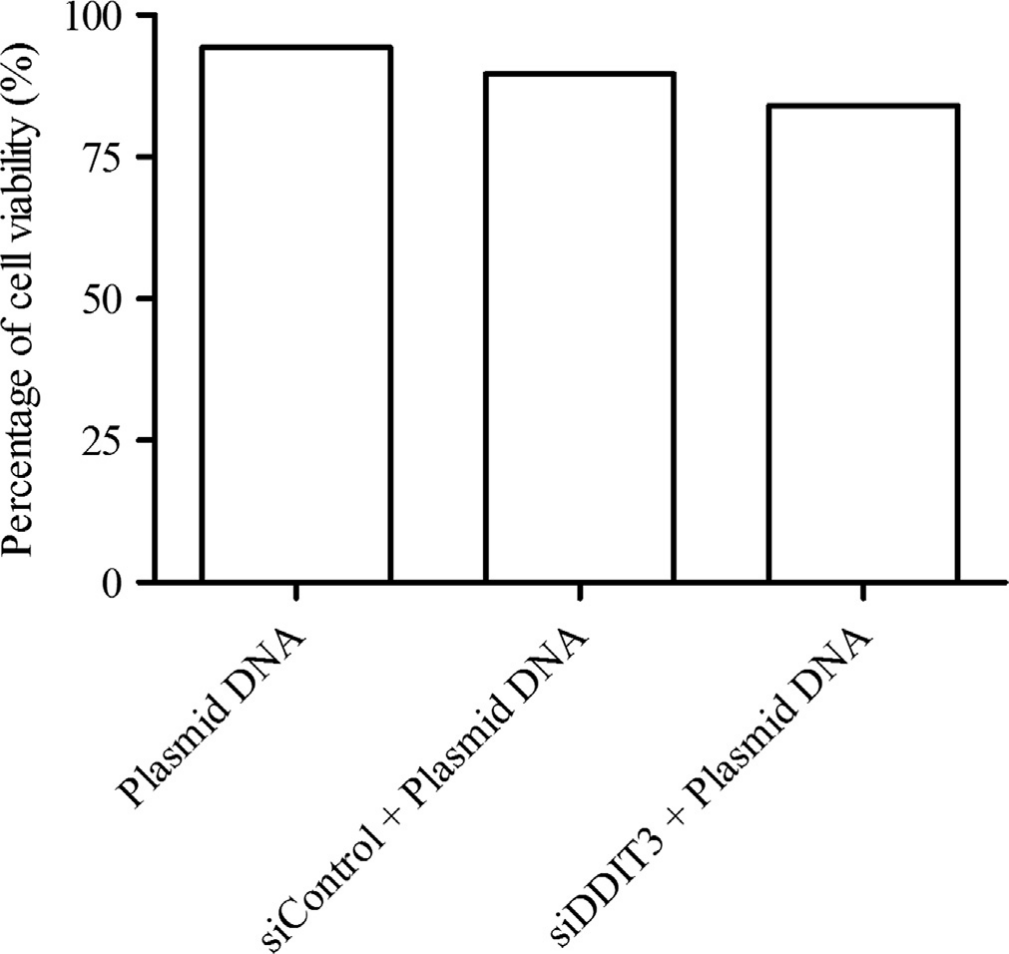
Cell viability of T-47D cells after transfection of either plasmid DNA (GFP-LC3 vector) or siDDIT3/siControl followed by plasmid DNA. Data are presented as mean replicates of a single independent experiment.

**Fig. 3:**
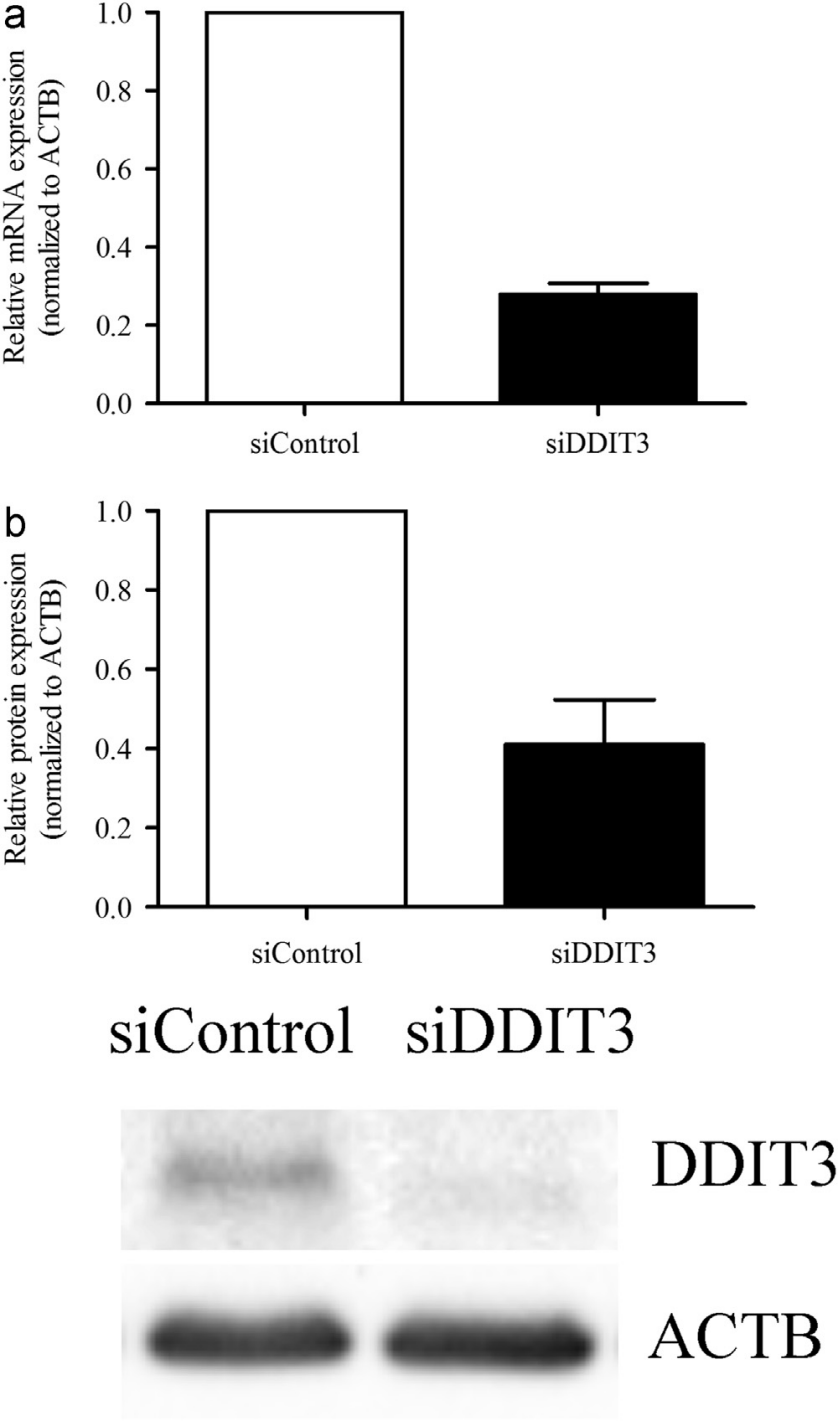
DDIT3-specific siRNA (siDDIT3) downregulated the mRNA and protein expression of DDIT3 in T-47D cells (A) mRNA and (B) protein expression of DDIT3 were determined using RT-qPCR and Western blot, respectively. Data are presented as mean ± SD of two independent experiments. Immunoblot image is representative.

Fig. 1 shows the cytotoxic parameters derived for 14-DDA in T-47D cells using the cell proliferation assay as described in materials and methods section. Fig. 2 shows the percentage of cell viability of double transfected T-47D cells as evaluated using trypan blue exclusion assay. The expression of DDIT3 in siDDIT3-transfected cells was evaluated using RT-qPCR and Western Blot analysis. Fig. 3 shows the relative gene (A) and protein (B) expressions of DDIT3 in siDDIT3-transfected cells as compared with control.

## 2 Experimental Design, Materials and Methods

### 2.1 Cell proliferation assay

T-47D cells were routinely cultured in RPMI 1640 medium supplemented with 10% (v/v) FBS. Briefly, cells were first seeded onto two 96-well plates (T_0_ and T_1_) and were allowed to grow for a day at 37 °C in a humidified incubator supplemented with 5% (v/v) CO_2_. Subsequently, cells in T_0_ plate were subjected to cell proliferation assay using fresh reduced serum medium (0.5% v/v FBS) at 24 h. On the same day, cells in T_1_ plate were cultured in reduced serum medium for 4 h and treated with various concentrations of 14-DDA. Control cells were treated with the same amount of vehicle (0.1% v/v DMSO). After 72 h, the cells were processed using CellTiter 96® Aqueous One Solution Cell Proliferation Assay (Promega, USA) following manufacturer׳s protocol. Briefly, 20 µl of MTS tetrazolium solution was added into each well and the plate was then incubated for 4 h and the absorbance was read at 490 nm using Envision 2104 multilabel plate reader (Perkin Elmer, USA). Percentages of growth (PG) were calculated using the formula as described by Boyd and co-workers [2]. PG were then plotted against concentration of 14-DDA and dose response curve were generated using GraphPad version 5.00 for Windows (GraphPad Software, USA) and interpolation points at PG of +50, 0 and −50% were determined. GI_50_ is the concentration of which PG is +50, indicating the increase in the number of cells in the test well is only 50% as compared with the control well. Total growth inhibition or TGI is the concentration indicating PG is zero and LC_50_ is the lethal concentration of which the PG is −50, indicating that the number of cells in the test well at the end of the incubation period is half the number of cells in the well at T_0_.

### 2.2 Cell viability and expression of DDIT3 in siDDIT3-transfected T-47D cells

The effects of double transfection (siRNA transfection followed by plasmid DNA transfection) on cell viability were evaluated using trypan blue exclusion test. Briefly, for double transfection, T-47D cells were transfected with either 30 nM of ON-TARGETplus™ SMARTpool DDIT3 siRNA (siDDIT3) or ON-TARGETplus™ Non-targeting Pool siRNA (siControl) (Dharmacon, USA) for 48 h (optimized condition) followed by plasmid DNA (GFP-LC3 vector) for 24 h. For single transfection, T-47D cells were transfected with plasmid DNA (GFP-LC3 vector) alone. Trypan blue exclusion test was then carried out to determine the effect of transfection on cell viability. Equal parts of 0.4% (v/v) trypan blue and cell suspension were mixed and incubate for several minutes at room temperature. A drop of the trypan blue/cell mixture was then placed on a hemacytometer and subsequently, stained (non-viable) and unstained cells were calculated.

To determine the siDDIT3 silencing effects in T-47D cells, the mRNA and protein expression of DDIT3 were evaluated in siDDIT3-transfected cells. Briefly, cells were first transfected with 30 nM of ON-TARGETplus™ SMARTpool DDIT3 siRNA (siDDIT3) or ON-TARGETplus™ Non-targeting Pool siRNA (siControl) (Dharmacon, USA) using Lipofectamine® RNAiMAX Reagent (Invitrogen, USA) for 72 h. Total RNA was then isolated as described previously [1]. Primers for DDIT3 were designed using Beacon Designer 7.80 (Premier Biosoft International, USA). PCR amplification was carried out with the following protocol: cycle 1 (cDNA synthesis) of 10 min at 50.0 °C; cycle 2 (reverse transcriptase inactivation) of 5 min at 95.0 °C; cycle 3 (PCR) of 10 s at 95.0 °C followed by 30 s at 54.1 °C repeated for 40 cycles and finally, cycle 4 (melt curve analysis), consisting of 5 s at 65–95 °C for 60 cycles, increasing by 0.5 °C for each cycle. Calculation of relative gene expression was according to the Pfaffl mathematical model [3].

Total cellular protein was isolated using ProteoJET™ Mammalian Cell Lysis Reagent (Fermentas, Canada) as described previously [1]. Briefly, the proteins were first separated by SDS-PAGE and then transferred to PVDF membrane. After blocking, the membranes were probed with DDIT3 antibodies (Cell Signaling Technology, USA) overnight and then incubated with IgG HRP-linked secondary antibodies for 3 h. The signal was detected using ECL™ Western Blotting Detection Reagents (Amersham Biosciences, UK) and imaged on ChemiDoc™ XRS Imaging System (Bio-Rad Laboratories, USA). Densitometric quantification was performed using Quantity One^®^ 1-D Analysis Software (Bio-Rad Laboratories, USA). Relative protein expression was derived by was normalizing DDIT3 against ACTB expression.
